# Smart thyroid-regulated drug delivery systems: a review of intelligent biosensor-integrated therapeutics for precision thyroxine replacement

**DOI:** 10.3389/fendo.2026.1843556

**Published:** 2026-07-02

**Authors:** Rahul Ingle, Gajanan M. Sonwane

**Affiliations:** 1 Department of Pharmaceutical Chemistry, Datta Meghe College of Pharmacy, Datta Meghe Institute of Higher Education and Research (Deemed to be University), Sawangi (M), Wardha, Maharashtra, India; 2 Department of Drug Discovery, Morepen Proprietary Drug Research Pvt Ltd, Hyderabad, Telangana, India

**Keywords:** artificial intelligence, biosensor, closed-loop drug delivery, levothyroxine, MEMS micro-pump, precision medicine, thyroid hormone replacement, thyroidectomy

## Abstract

Levothyroxine (L-T4) replacement therapy has remained the standard treatment for post-thyroidectomy hypothyroidism since the mid-twentieth century. The drug works, but the delivery method has a basic flaw: it cannot adjust. A patient swallows the same dose every morning regardless of whether she is pregnant, fighting a fever, fasting, or sleeping. The healthy thyroid gland, by contrast, constantly tunes its output in response to signals from the hypothalamic-pituitary-thyroid (HPT) axis, adjusting on a timescale of minutes to hours. This mismatch between static pills and dynamic physiology leaves most thyroidectomized patients oscillating between mild over- and under-replacement for much of their lives. Recent meta-analyses confirm that only about 34% of these patients hit target euthyroidism at their first post-operative follow-up. The consequences are not trivial: cardiac arrhythmias, bone mineral loss, cognitive fog, metabolic disruption, and impaired fertility all track with even mild thyroid hormone imbalance. This review makes the case that we now have the pieces to build something better. Advances in nanomaterial-based biosensors already permit picomolar-range detection of TSH, free T3, and free T4. Machine learning pharmacokinetic models predict individual L-T4 requirements with R-squared values exceeding 0.85. The artificial pancreas has demonstrated that closed-loop drug delivery can work safely in humans at scale. We propose an Intelligent Medicine Bot (IMB): a wearable or implantable closed-loop platform that continuously senses thyroid hormone levels, computes optimal doses through adaptive AI algorithms, and delivers precisely titrated levothyroxine through a MEMS micro-pump. We review the clinical problem in detail, survey the enabling technologies, present the system architecture, and outline a phased development roadmap toward clinical translation.

## 1 Introduction

### 1.1 The thyroid gland: functions and regulation

The thyroid gland sits in the anterior neck, weighs about 20 grams, and punches well above its weight. Through two hormones, thyroxine (T4) and triiodothyronine (T3), it regulates basal metabolic rate, heart rhythm, body temperature, brain function, bone turnover, and lipid metabolism. Regulation happens through the HPT axis: the hypothalamus releases thyrotropin-releasing hormone (TRH), which tells the pituitary to secrete thyroid-stimulating hormone (TSH), which tells the thyroid to produce T4 and T3. When circulating hormone levels are adequate, the hypothalamus and pituitary dial back. The loop is continuous and precise (1, 3).

Total thyroidectomy, the complete surgical removal of the gland, is performed for thyroid cancer, large goiter, severe Graves’ disease, and destructive infections. Roughly 1.5 million thyroidectomies are performed worldwide each year (26). Every one of those patients becomes permanently hypothyroid and needs lifelong hormone replacement.

### 1.2 The global burden and current standard of care

Hypothyroidism in general affects up to 5% of the global population (5). Levothyroxine is consistently among the three most-prescribed medications on the planet. Yet despite its ubiquity, the therapy fails to achieve stable hormone levels in a disturbingly large fraction of patients. A 2025 systematic review and meta-analysis in the Journal of Clinical Endocrinology and Metabolism, covering 11 studies and 2,577 patients, found that the pooled euthyroidism rate at first follow-up after total thyroidectomy was just 33.9% (2). Separate data suggest that at any given time, roughly half of all patients on L-T4 have TSH values outside the optimal range (24).

### 1.3 Why current tablets cannot solve this

A levothyroxine tablet delivers a fixed quantity of synthetic T4. The patient swallows it in the morning, absorption occurs over two to four hours through the proximal gut, and the drug produces a peak in circulating T4 followed by a gradual decline. This pharmacokinetic profile bears little resemblance to the steady, demand-responsive output of a functioning thyroid gland. The tablet cannot tell whether the patient slept poorly, skipped breakfast, caught a cold, or started an exercise program. It delivers the same dose regardless, and the body is left to cope with whatever mismatch results (1, 4).

**Figure 1:**
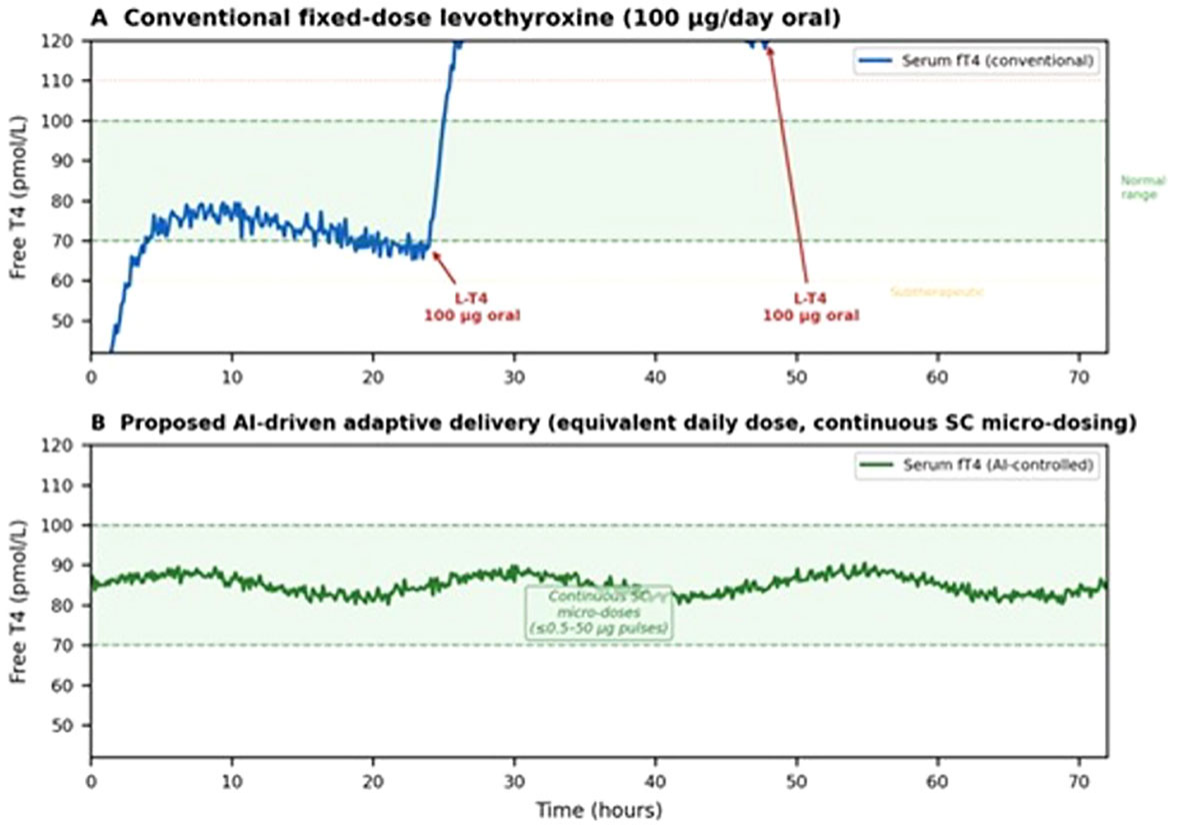
Simulated pharmacokinetic profiles comparing conventional fixed-dose L-T4 therapy **(A)** with a proposed AI-driven adaptive delivery system **(B)**. In Panel **(A)**, a fixed oral dose of 100 µg levothyroxine is administered once daily (red arrows), representing a typical post-thyroidectomy replacement dose for a 60–70 kg patient (approximately 1.5 µg/kg/day). The characteristic supraphysiological peak at 2–4 hours post-dose and subtherapeutic trough at 18–24 hours are evident. In Panel **(B)**, the same total daily levothyroxine exposure is delivered as continuous subcutaneous micro-doses (0.5–50 µg pulses) by the proposed AI-controlled system, maintaining serum free T4 within the normal reference range (green shaded zone, 70–100 pmol/L). Simulated profiles based on published pharmacokinetic parameters (4, 21). Note: actual replacement doses vary widely between patients (typically 75–200 µg/day) depending on body weight, residual thyroid function, and individual absorption.

Absorption itself varies by 60% to 80% of the administered dose depending on gastric pH, food intake, concurrent medications (calcium, iron, proton pump inhibitors), and gastrointestinal pathology (21). Once absorbed, T4 must be converted to the active hormone T3 by deiodinase enzymes, a step that varies between individuals based on genetic polymorphisms in DIO1 and DIO2 (20). The consequence is a therapy characterized by supraphysiological peaks 2–4 hours after ingestion and subtherapeutic troughs 18–24 hours later (**Figure 1**).

### 1.4 Scope and aims of this review

This review has three aims. First, we examine the clinical evidence for why fixed-dose L-T4 therapy consistently underperforms, including the multi-organ consequences of imprecise replacement. Second, we survey the technologies that are maturing in parallel – thyroid hormone biosensors, machine learning dosing models, and miniaturized drug delivery devices – and assess their readiness levels. Third, we propose a specific integration of these technologies into a closed-loop Intelligent Medicine Bot (IMB) and outline the development pathway toward clinical translation. Throughout, we identify the key controversies in the field, the current research gaps, and the potential developments that could reshape thyroid hormone replacement over the coming decade.

## 2 Clinical consequences of imprecise thyroid hormone replacement

**Figure 2:**
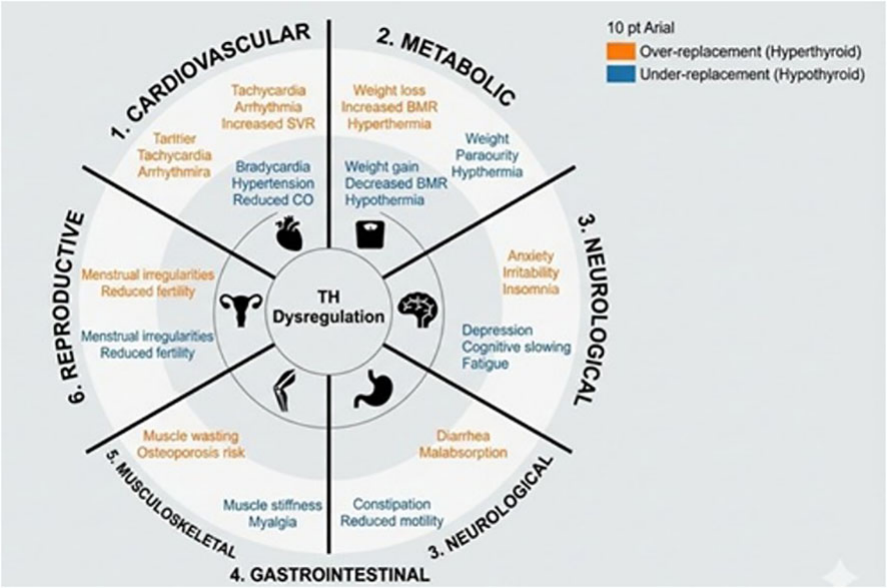
Multi-organ consequences of thyroid hormone dysregulation. Each organ system is affected differently by over- and under-replacement, creating a narrow therapeutic window that static dosing struggles to maintain.

The downstream effects of even mild thyroid hormone imbalance are wide-ranging and clinically documented. Over-replacement pushes patients toward subclinical hyperthyroidism; under-replacement pushes them toward subclinical hypothyroidism. Neither is benign. The ATA guidelines (3), the ETA guidelines (20), and multiple large observational cohort studies have established clear associations between thyroid hormone dysregulation and adverse outcomes across organ systems (4, 19) (**Figure 2**; **Table 1**).

**Table 1:** Multi-organ consequences of thyroid hormone dysregulation in thyroidectomized patients.

Organ system	Over-replacement	Under-replacement	Clinical significance
Heart	Atrial fibrillation (3x risk), tachycardia, QT dispersion, angina	Diastolic hypertension, dyslipidemia, accelerated atherosclerosis	Elevated cardiovascular mortality in both directions
Skeleton	Accelerated bone turnover, reduced BMD, fracture risk (2x in postmenopausal women)	Altered calcium metabolism, impaired remodeling	Long-term skeletal morbidity from chronic imbalance
Brain	Anxiety, tremor, insomnia, difficulty concentrating	Fatigue, depression, cognitive slowing, memory impairment	Quality of life impairment; reduced occupational function
Metabolism	Weight loss, heat intolerance, insulin resistance	Weight gain, cold intolerance, dyslipidemia	Bidirectional metabolic disruption; diabetes risk
Reproductive	Menstrual irregularities, reduced fertility	Infertility, pregnancy loss, fetal neurodevelopmental harm	Particularly dangerous in women of reproductive age
Muscle	Proximal myopathy, exercise intolerance	Myalgia, cramps, elevated CK	Functional impairment in daily activities

BMD, bone mineral density; CK, creatine kinase. Data compiled from ATA guidelines (3), ETA guidelines (20), and multiple observational cohort studies (4, 19).

## 3 Current dosing strategies and their limitations

### 3.1 Static dosing approaches

Fixed-dose prescribing (typically 100–150 micrograms per day) is the simplest but crudest method: it ignores all individual variation. Weight-based dosing at 1.6 micrograms per kilogram is incrementally better, but the assumed linear relationship between body weight and L-T4 requirement breaks down in obese patients, elderly patients, and those with absorption problems (28). Neither method accounts for concurrent medications, dietary factors, circadian variation, or dynamic physiological states such as illness, pregnancy, or stress (1, 21).

### 3.2 Predictive models and machine learning

**Figure 3:**
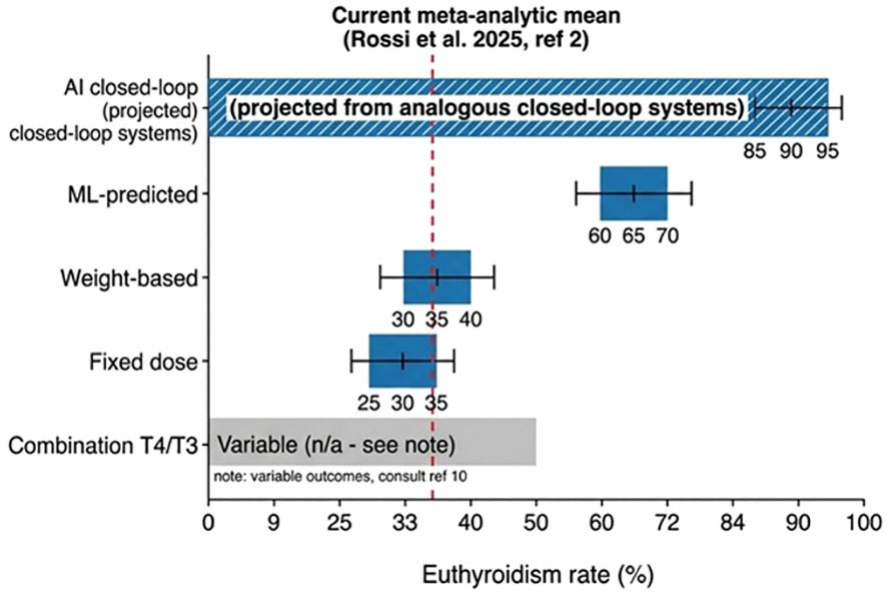
Estimated and projected euthyroidism achievement rates across dosing strategies. The dashed red line marks the current pooled meta-analytic mean of 33.9% (2). The AI closed-loop projection is derived from artificial pancreas performance data in type 1 diabetes.

Several groups have developed multivariate algorithms and machine learning models that incorporate age, BMI, comorbidities, concurrent medications, and serum albumin levels. A 2025 study found that an Extra Trees Regressor model predicted L-T4 dosage with an R-squared of 0.87 (6). A separate Poisson regression model incorporating seven variables predicted 60.9% of doses correctly in a cohort of 598 patients who attained euthyroidism after thyroidectomy (2) (**Figure 3**; **Table 2**). These models are impressive as starting-point predictions, but they are inherently retrospective and static: they estimate an initial dose and then stop. They do not adjust therapy as conditions change.

**Table 2:** Comparative analysis of thyroid hormone dosing strategies.

Strategy	Euthyroidism rate	Advantage	Limitation	Adaptive?
Fixed dose	25–35%	Simple, low cost	Ignores all individual variation	No
Weight-based (1.6 µg/kg)	30–40%	Accounts for body size	Inaccurate in obesity, old age, malabsorption	No
ML-predicted	60–70%*	High accuracy initial dose	Static; no real-time feedback	Predictive only
Combination T4/T3	Variable	Addresses poor T4-to-T3 converters	T3 peaks/troughs; complex regimen	No
AI closed-loop (proposed)	85–95%†	Real-time adaptive; personalized	Under development; device cost	Fully adaptive

*Based on retrospective validation studies (2, 6). †Projected from analogous closed-loop systems in diabetes (artificial pancreas data). ML, machine learning.

### 3.3 Alternative formulations and delivery routes

Combination T4/T3 therapy aims to address the problem that some patients are poor peripheral converters of T4 to T3. However, T3 has a short half-life (about one day), producing its own peaks and troughs. The ATA guidelines concluded that there is no consistently strong evidence for the superiority of combination therapy over levothyroxine monotherapy (3). Desiccated thyroid extract has variable potency and is difficult to titrate. Liquid levothyroxine formulations improve absorption consistency compared to tablets, particularly in patients with gastrointestinal disorders (1, 18).

A notable development is XP-8121, a once-weekly subcutaneous levothyroxine injection currently in Phase 2 clinical trials. Pharmacokinetic modelling supports a conversion factor of approximately 4x the daily oral dose for equivalent weekly exposure (17). This approach addresses adherence and absorption variability but still delivers a fixed, pre-determined dose that cannot respond to real-time physiological changes.

### 3.4 Schools of thought on optimal replacement

The field remains divided on several questions. Some endocrinologists advocate strict TSH-based dosing, aiming to normalize TSH as the sole therapeutic target. Others argue that patient symptoms and quality of life should drive dose adjustments, even when TSH falls within the reference range (3, 27). A third perspective, supported by growing molecular evidence, holds that thyroid hormone replacement should aim to restore tissue-level hormone action, which may require different circulating hormone concentrations in different individuals depending on deiodinase polymorphisms and receptor sensitivity (20). These unresolved debates directly inform the rationale for an adaptive, personalized delivery system: if the optimal dose varies not just between patients but within the same patient over time, then static dosing is inherently unable to solve the problem.

## 4 Proposed solution: the intelligent medicine bot

### 4.1 Conceptual framework

**Figure 4:**
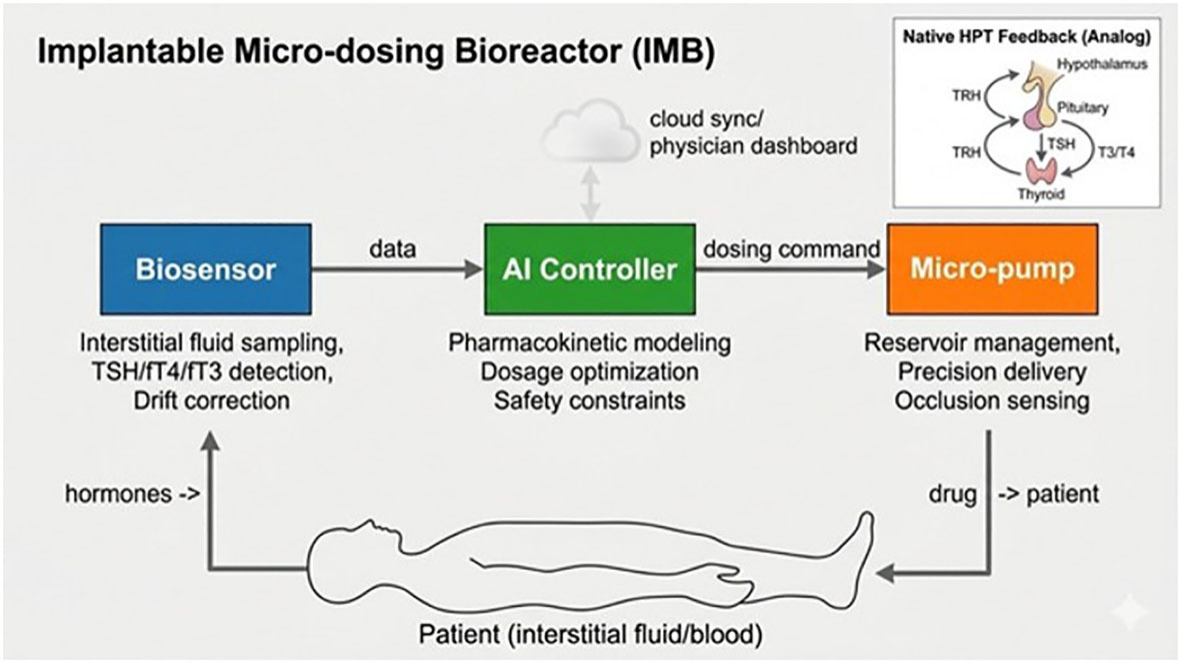
System architecture of the proposed intelligent medicine bot (IMB). The biosensor module continuously monitors thyroid hormones via interstitial fluid sampling. Data flows to the AI controller, which computes optimal dosing and sends commands to the micro-pump. The cloud database enables physician remote monitoring and algorithm updates. The closed feedback loop mimics the native HPT axis.

We propose a closed-loop system that operates on the same logic as the HPT axis: continuous sensing, intelligent processing, adaptive response. The system has three tightly coupled modules: a biosensor, an AI controller, and a micro-pump delivery unit (**Figure 4**).

The concept is not without precedent. Closed-loop insulin delivery systems (artificial pancreas devices) are FDA-approved and commercially available from Medtronic, Tandem, and others. They combine continuous glucose monitors with algorithmic controllers and insulin pumps, and they have measurably improved glycemic control for hundreds of thousands of diabetic patients (13). The thyroid version is harder in some respects – thyroid hormones act on a timescale of days rather than minutes, and the biosensors are less mature – but the architectural blueprint is proven. A 2025 review in Nature Reviews Bioengineering surveyed the entire landscape of smart closed-loop drug delivery systems and identified AI-enhanced control algorithms and synthetic biology integration as the key frontiers (13). A March 2026 review in Nature examined intelligent and miniaturized drug delivery devices, emphasizing that real-time sensing and adaptive control are now technically achievable (14).

### 4.2 Module 1: the biosensor

The sensor module must measure TSH, free T4, and ideally free T3 in real time. Several technologies are converging toward this goal (**Table 3**).

**Table 3:** Emerging biosensor technologies for thyroid hormone monitoring.

Technology	Analyte	LOD	Speed	Wearable?	TRL	Ref.
Gold NP lateral flow	TSH	0.017 µIU/mL	<25 min	Moderate	4–5	(7)
Graphene nanocomposite	fT3	13.7 nM	<10 min	High	3–4	(8)
Microneedle + MIP	fT4	Sub-nM	Continuous	Very high	2–3	(9)
Intein-mediated	T3/T4	Sub-µM	~30 h	Low	2	(10)
ThyroSense POC	TSH	0.2 mIU/L	5 min	High	4	(11)
THYROSCOPE	HR proxy	N/A	Continuous	High	5–6	(12)

LOD, limit of detection; NP, nanoparticle; MIP, molecularly imprinted polymer; POC, point-of-care; HR, heart rate; TRL, technology readiness level.

Nanomaterial-based electrochemical sensors using gold nanoparticles, graphene derivatives, and carbon nanotubes can detect TSH with limits as low as 0.017 microIU/mL (7). For context, the clinically relevant TSH range is 0.4–4.0 mIU/L. A graphene nanocomposite biosensor demonstrated selective fT3 detection at nanomolar concentrations (8). A molecularly imprinted polymer (MIP) sensor paired with hollow microneedles, under development by Integrated Molecular Innovations, samples interstitial fluid from the dermis and measures free T4 continuously without blood draws (9). A separate research group built intein-mediated biosensors based on chimeric proteins that fluoresce proportionally to T3/T4 concentration, with an R-squared of 0.99 against known standards (10).

The ThyroSense prototype, presented at the 2025 ESE/ESPE congress, achieved a detection limit of 0.2 mIU/L for TSH with a coefficient of variation of 10.7–13.0% and results in under five minutes, using a disposable cartridge costing less than five pounds (11). The THYROSCOPE system takes an indirect approach, correlating continuously monitored heart rate data from commercial wearables with thyroid hormone status using machine learning classification (12).

### 4.3 Module 2: the AI controller

The computational core handles three tasks: signal processing, pharmacokinetic modelling, and dose optimization. Raw sensor data arrives noisy; edge computing on a local microcontroller filters artefacts and extracts trends. The controller maintains a digital twin of the individual patient’s thyroid hormone pharmacokinetics: a computational model, initially trained on population-level data, that continuously refines itself using the patient’s own response history. Ensemble machine learning methods (Extra Trees, gradient boosting) already predict L-T4 requirements with R-squared values above 0.85 (6). Layered on top is a reinforcement learning algorithm that learns a personalized dosing policy by optimizing for time-in-target-range while penalizing excursions.

The ML architecture integrates multiple inputs beyond hormone levels: heart rate, skin temperature, activity level (from accelerometer data), heart rate variability as a stress proxy, time of day, and recent meal timing. An LSTM network captures temporal patterns in hormone kinetics. A gradient boosting model handles event-based predictions. Bayesian optimization handles dose titration. The controller produces an immediate dose command, a 6–12 hour forecast, alert flags for the patient and physician, and a weekly trend summary.

Safety is non-negotiable. The architecture includes hard-coded dose ceilings that the algorithm cannot override, anomaly detection that flags unusual readings for human review, a physician dashboard with remote monitoring and manual override capability, and complete audit logging of all dose decisions.

### 4.4 Module 3: the micro-pump delivery unit

The delivery device is a miniaturized subcutaneous unit (approximately 5 × 3 × 1 cm) containing a 7–14 day levothyroxine reservoir, a MEMS micropump capable of 0.5–50 microgram pulses with 2–5% precision, a wireless microcontroller, and a rechargeable battery with transcutaneous charging. The device receives dosing commands from the AI controller and delivers micro-doses throughout the day, producing a stable plasma concentration profile rather than the saw-tooth curve of oral dosing.

Several published findings support this design. The subcutaneous levothyroxine formulation XP-8121, currently in Phase 2 clinical trials, demonstrates that subcutaneous L-T4 delivery produces sustained, stable hormone exposure (17). A 2025 report in Nature described a battery-free nanofluidic delivery patch for internal organs (14). MEMS micro-pump technology is already commercially deployed in insulin pump systems.

## 5 Enabling precedents: closed-loop systems that already work

The strongest argument for feasibility is that the hardest version of this problem has already been solved in a different organ system (**Table 4**). Insulin has a half-life of minutes and a narrow therapeutic window; errors in dosing cause immediate, life-threatening hypoglycemia. Levothyroxine has a half-life of seven days and a wider therapeutic window. If a closed-loop system can safely manage insulin delivery, a thyroid hormone version faces a less demanding pharmacokinetic challenge.

**Table 4:** Precedent closed-loop systems and their relevance to thyroid application.

System	Disease	Biomarker	Achievement	Thyroid relevance
Artificial pancreas	Type 1 diabetes	Glucose	FDA-approved; 70%+ time-in-range; commercial	Direct architectural blueprint; regulatory precedent
Aptamer-based CLS	Cancer (doxorubicin)	Drug level	First closed-loop chemo *in vivo* (rabbits)	Proves real-time drug feedback is feasible
XP-8121 SC L-T4	Hypothyroidism	TSH, fT4	Phase 2: stable weekly SC dosing	Validates subcutaneous levothyroxine route
ThyroSense POC	Hypothyroidism	TSH	5-min home TSH at <£5/test	Affordable frequent monitoring possible
THYROSCOPE	Thyrotoxicosis	Heart rate	ML-based thyroid status from wearables	Non-invasive continuous surrogate monitoring

CLS, closed-loop system; SC, subcutaneous; POC, point-of-care; ML, machine learning. References 7, 11–17:.

## 6 Development roadmap

**Figure 5:**
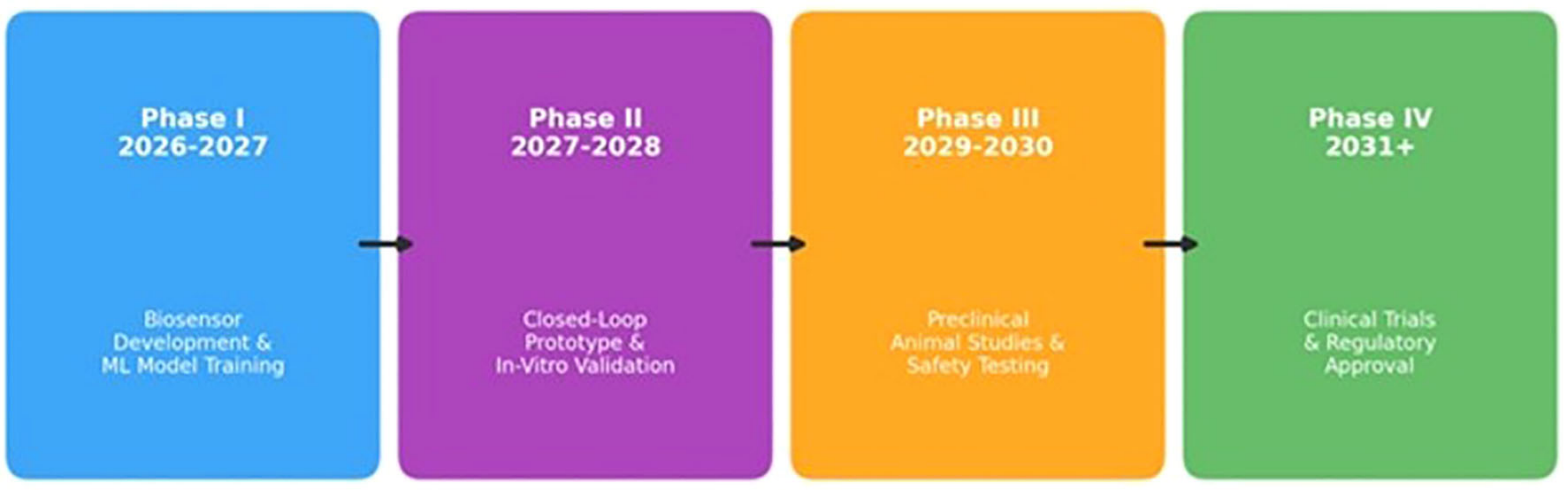
Phased development roadmap for the intelligent medicine bot.

Feasibility. Phase 1 (Years 1–2): Develop and validate the core biosensor for TSH and fT4 in interstitial fluid (**Figure 5**). Demonstrate continuous operation for at least seven days without recalibration. Train initial ML pharmacokinetic models on retrospective clinical data. Perform biocompatibility testing per ISO 10993.

Integration. Phase 2 (Years 2–3): Assemble the integrated prototype linking biosensor, AI controller, and micro-pump. Validate closed-loop performance *in vitro* using artificial tissue models. Test in porcine thyroidectomy models. Submit FDA Investigational Device Exemption (25).

Clinical trials. Phase 3 (Years 3–5): Phase I safety trial in healthy volunteers. Phase II dose-finding in thyroidectomized patients. Phase III randomized controlled trial comparing IMB to standard oral L-T4. Primary endpoints: percentage of time within target TSH/fT4 range, quality of life scores, adverse events.

Commercialization. Phase 4 (Years 5–6): FDA Pre-Market Approval submission. CE Mark for EU. GMP manufacturing scale-up. Initial commercial launch through specialized endocrinology centers, then broader rollout.

## 7 Discussion

### 7.1 Key challenges and mitigation strategies

Several formidable technical challenges must be addressed before the IMB can reach clinical use (**Table 5**). Biosensor drift due to biofouling and degradation of the recognition element remains a universal problem in implantable sensing (13, 15). Mitigation strategies include anti-fouling surface coatings, redundant sensor arrays, and auto-recalibration algorithms. Power consumption for continuous sensing and wireless communication must be minimized; ultra-low-power electronics and body-heat energy harvesting offer partial solutions, as demonstrated in existing continuous glucose monitors. Drug stability within the micro-pump reservoir is another concern; lyophilized levothyroxine formulations with on-demand reconstitution and nitrogen-purged headspace may address this (18).

**Table 5:** Key technical and regulatory challenges with proposed mitigations.

Domain	Challenge	Proposed mitigation	Current status
Biosensor drift	Biofouling and signal degradation over weeks	Anti-fouling coatings; redundant sensor arrays; auto-recalibration	TRL 2–3; active research
Power	Continuous sensing drains batteries	Ultra-low-power electronics; body-heat energy harvesting	TRL 3–4; demonstrated in CGMs
Drug stability	L-T4 may degrade in reservoir	Lyophilized formulation with on-demand reconstitution	TRL 3; feasibility stage
AI safety	Wrong dose computation risk	Hard-coded dose limits; anomaly flags; physician override	TRL 4–5; proven in diabetes
Regulatory	Novel combination product category	Follow artificial pancreas pathway; early FDA/EMA dialogue	Precedent exists
Cost	High device cost vs. cheap tablets	Modular design; reusable controller with disposable sensor	TRL 1–2; design phase

TRL, technology readiness level; CGM, continuous glucose monitor.

### 7.2 Controversies and competing perspectives

Not everyone in the thyroid field will agree that a closed-loop device is necessary. One school of thought holds that the current failure rates of L-T4 therapy are primarily a problem of implementation, not technology: if clinicians simply monitored patients more frequently, adjusted doses more promptly, and paid closer attention to absorption-altering factors, outcomes would improve without the complexity and cost of an implantable device. There is some merit to this view; adherence to monitoring guidelines is poor in routine practice, and better implementation of existing protocols would certainly help.

A second perspective argues that the LT4/T3 combination therapy debate remains unresolved, and that the real gap in current therapy is not dose timing but the failure to replace T3 directly in patients with deiodinase polymorphisms (3, 20). From this standpoint, a device that delivers only L-T4, however precisely, still misses the point for a subset of patients. A future iteration of the IMB could potentially incorporate T3 co-delivery, but this adds pharmacokinetic complexity given T3’s much shorter half-life.

A third, pragmatic objection concerns cost-effectiveness. The majority of hypothyroid patients are adequately managed on inexpensive oral L-T4 at a cost of a few thousand rupees per year. An IMB costing 150,000–300,000 rupees for the implant will not be cost-effective for patients who are well-controlled on tablets (**Table 6**). The strongest initial use case is therefore patients who are demonstrably poorly controlled despite optimized oral therapy: those with significant malabsorption, unstable physiology (e.g. recurrent illness), pregnancy-related fluctuations, or post-thyroidectomy cancer patients requiring tight TSH suppression.

**Table 6:** Comparative cost analysis: conventional therapy versus intelligent medicine bot.

Cost category	Conventional therapy	IMB system
Annual drug cost	₹2,000–5,000	₹2,000–5,000 (same drug)
Laboratory monitoring	₹6,000–15,000/year	₹2,000–5,000/year (fewer visits)
Device implant	N/A	₹150,000–300,000 (one-time)
Sensor replacement	N/A	₹30,000–60,000/year
Complication cost avoided	Significant (AF, fractures)	Substantially reduced

Improved symptom control and reduced long-term complications could yield an estimated 0.5–1.5 additional QALYs over 10 years, potentially making the system cost-effective under WHO-CHOICE thresholds for appropriately selected patients. AF, atrial fibrillation; QALY, quality-adjusted life year.

### 7.3 Current research gaps

Several critical gaps must be filled before a thyroid closed-loop system becomes viable:

First, no continuous thyroid hormone biosensor has been validated in human interstitial fluid over periods longer than a few hours. The published biosensor data come from bench-top studies using buffer solutions or spiked serum samples (7–11). Translating these to the complex, protein-rich environment of subcutaneous tissue is a major open question.

Second, the relationship between interstitial fluid thyroid hormone concentrations and plasma concentrations is not well characterized. Insulin closed-loop systems benefited from decades of research establishing the glucose kinetics between blood and interstitial fluid; equivalent data for T4 and T3 do not yet exist.

Third, the optimal control algorithm for thyroid hormone dosing has not been developed. The slow pharmacodynamics of levothyroxine (half-life of approximately seven days, with clinical effects manifesting over weeks) mean that standard proportional-integral-derivative controllers used in artificial pancreas systems would need fundamental modification. Model predictive control or reinforcement learning approaches may be better suited, but this remains an open research question.

Fourth, the regulatory pathway for a combination drug-device-AI product in thyroid disease has not been established. While the artificial pancreas provides precedent, thyroid-specific guidance from FDA or EMA does not yet exist (25).

### 7.4 Potential developments

Looking forward, several emerging technologies could accelerate the IMB’s development. Synthetic biology approaches could yield cell-based implants that produce thyroid hormones in response to TSH levels, potentially eliminating the need for an external drug reservoir entirely. Digital twin technology, in which a computational model of an individual patient’s physiology is continuously updated with real-world data, could enable predictive rather than merely reactive dose adjustment. Federated learning approaches could allow the AI controller to improve its dosing algorithms using data from thousands of patients while preserving individual privacy (14, 15).

If the IMB works for thyroid hormone replacement, the same architecture applies to multiple chronic diseases where static dosing fails and physiological demand is dynamic: adrenal insufficiency patients on hydrocortisone, Parkinson’s patients on levodopa, and growth hormone deficiency patients all face analogous problems (22, 23). The artificial pancreas proved the concept for one hormone; there is no inherent reason the platform cannot extend to others.

## 8 Conclusion

We have been giving thyroidectomized patients the same kind of therapy since 1949: a pill, once a day, same dose, regardless of what the body actually needs. It has saved millions of lives and improved millions more, and it deserves credit for that. But it is also, at a basic level, a blunt instrument being used for a precision job.

The pieces to build something better now exist. Biosensors can detect thyroid hormones at clinically useful concentrations. Machine learning models can predict individual dosing requirements with reasonable accuracy. Closed-loop control has been validated in analogous systems. Miniaturized drug delivery devices can administer precise, programmable doses. What remains is the work of integration: assembling these components into a cohesive, reliable, and affordable system, then proving that it works in patients.

That is not a trivial engineering challenge, but it is a tractable one. We have identified the key research gaps – interstitial fluid biosensor validation, T4/T3 kinetics characterization, control algorithm development, and regulatory pathway establishment – and proposed a phased roadmap to address them. The millions of post-thyroidectomy patients worldwide who spend their lives oscillating between mild over- and under-replacement deserve a therapy that matches the sophistication of the problem. The Intelligent Medicine Bot is a concrete proposal for that therapy, and its development should be pursued with both urgency and rigor.
